# Kir3 channel blockade in the cerebellar cortex suppresses performance of classically conditioned Purkinje cell responses

**DOI:** 10.1038/s41598-020-72581-8

**Published:** 2020-09-24

**Authors:** Fredrik Johansson, Germund Hesslow

**Affiliations:** Associative Learning Group, Department of Experimental Medical Science, Lund University, BMCF10, 22184 Lund, Sweden

**Keywords:** Classical conditioning, Cerebellum

## Abstract

In the eyeblink conditioning paradigm, cerebellar Purkinje cells learn to respond to the conditional stimulus with an adaptively timed pause in its spontaneous firing. Evidence suggests that the pause is elicited by glutamate released from parallel fibers and acting on metabotropic receptors (mGluR7) which initiates a delayed-onset suppression of firing. We suggested that G protein activation of hyperpolarizing K_ir_3 channels (or ‘GIRK’, G protein-coupled inwardly-rectifying K^+^ channels) could be part of such a mechanism. Application of the K_ir_3 antagonist Tertiapin-LQ locally in the superficial layers of the cerebellar cortex in decerebrate ferrets suppressed normal performance of Purkinje cell pause responses to the conditional stimulus. Importantly, there was no detectable effect on spontaneous firing. These findings suggest that intact functioning of K_ir_3 channels in the cerebellar cortex is required for normal conditioned Purkinje cell responses.

## Introduction

Response timing, a critical aspect of most behaviors, can be studied in eyeblink conditioning. If a conditional stimulus (CS), repeatedly precedes an unconditional blink-eliciting stimulus (US), at a fixed delay, it acquires the ability to elicit a conditional blink response (CR) that peaks near the expected time of the US^1–3^. This learning depends on the cerebellar cortex^4^ where Purkinje cells receive information about the CS and US via mossy/parallel fibers and climbing fibers, respectively^5–8^.

Purkinje cells are inhibitory neurons that are driven by intrinsic pacemaker mechanisms to fire at high rates of 50–100 Hz in vivo^9^. Initially, presentations of the CS have either no effect or an excitatory effect on the Purkinje cells’ firing. However, during conditioning with sensory^10–12^, direct mossy fiber^13^, or direct parallel fiber^14^ stimulation as CS, Purkinje cells learn to suppress their firing in response to the CS. This firing “pause”, a “Purkinje cell CR”, removes inhibition of cerebellar nuclear cells, which increase their firing to generate the overt, conditional blink^15,16^. The pause responses display almost all known features, including adaptive timing, of the overt CR^17^. Overt CRs reach their maximum amplitude just before the anticipated onset of the US and the Purkinje cell CRs reach their maximum a few tens of milliseconds earlier^11,14,18^, consistent with delays in the motor pathways^19^.

The common hypotheses for the underlying learning mechanism have focused on network mechanisms thought to generate time-varying activation of different parallel fibers during the CS (reviewed in^20,21^). This could allow for selective long-term depression of those parallel fiber-to-Purkinje cell synapses that are most active near the onset of the climbing fiber US. Resulting net inhibition from GABAergic interneurons would then inhibit the Purkinje cell around the time of the US.

However, Purkinje cells learn adaptively timed CRs to a CS consisting of a uniform train of stimuli repetitively applied to the same parallel fibers, i.e. when the upstream network is bypassed^21^. Furthermore, both their GABA_A_^14^ and AMPA-kainate receptors^22^ can be blocked without disrupting the CR. Instead, the evidence suggests that timed Purkinje cell CRs are mediated by a mechanism intrinsic to the Purkinje cell, which is activated by parallel fiber release of glutamate acting on mGluR7^22^.

Very little is known about mGluR7 actions in the Purkinje cell but the metabotropic nature of the activation of the Purkinje cell CR suggests that some non-standard type of ion channel might be involved in generating a delayed hyperpolarization.

Gβγ activation of hyperpolarizing K_ir_3 channels (or ‘GIRK’, G protein-coupled inwardly-rectifying K^+^ channels)^23,24^ is a plausible candidate mechanism for the following reasons. Various K_ir_3 channel subunits are expressed and form multiple heteromeric combinations in the Purkinje cell^25^. The differences in subunit composition of the channels confer a variety of temporal properties^26^ to the channel complex. This, together with the fact that the temporal dynamics of the G protein actions are controlled by the regulator of G protein signaling (RGS) family^27^, would seem to make these channels sufficiently flexible and well suited for generating timed responses in the range of hundreds of milliseconds.

Here, we aimed to test whether the G-protein gated K_ir_3 channels are required for normal performance of Purkinje cell CRs by applying the antagonist Tertiapin_LQ_ close to conditioned Purkinje cells in decerebrate ferrets. This drug was selected for its antagonist effect on different Kir3 subunit combinations and while it is also a potent Kir1 channel blocker, Kir1 is not present in the cerebellar cortex.

## Results

**Figure 1:**
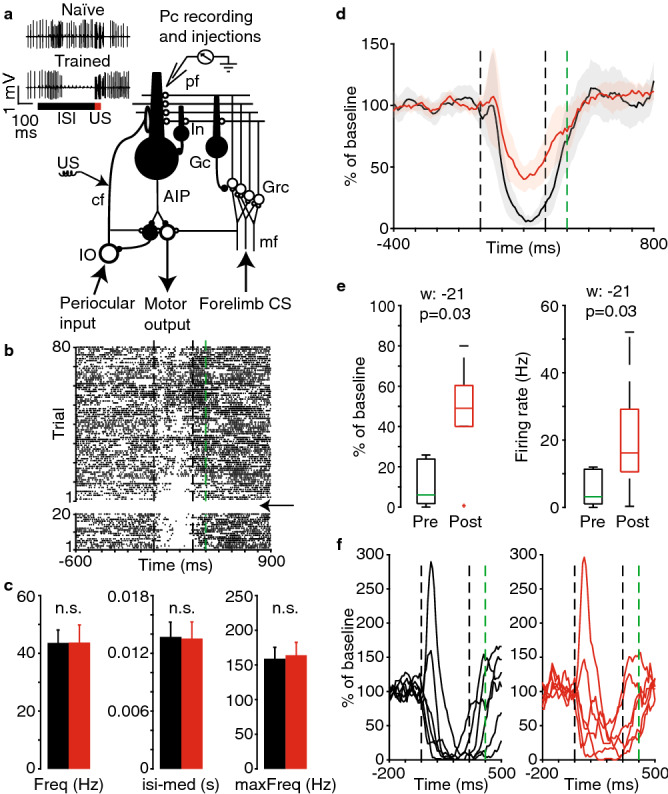
Effects of the K_ir_3 antagonist Tertiapin_LQ_ on conditional Purkinje cell responses. (**a**) Simplified neural circuitry with stimulation, recording and injections sites. Figure also shows a typical example of naïve and conditional Purkinje cell responses to a forelimb CS. *CS* conditional stimulus, *US* unconditional stimulus, *ISI* Interstimulus interval, *IO* inferior olive, *cf* climbing fiber, *mf* mossy fiber, *Pc* Purkinje cell, *pf* parallel fibers, *Grc* granule cell, *AIP* anterior interpositus nucleus. (**b**) Raster plot of a Purkinje cell’s responses to the CS before (20 trials) and after (80 trials) injection of 5 μM Tertiapin_LQ_. Arrow indicates injection. (**c**) Statistics of spontaneous firing before (black) and after (red) injection of 5 μM Tertiapin_LQ_. (**d**) Population average (n = 6) of Purkinje cell responses to the CS before (black) and after (red) injection of 5 μM Tertiapin_LQ_. Black dashed lines indicate the 300 ms CS-US interval used during conditioning (no US on test trials). Green dashed line indicates the end of the 400 ms CS. The traces represent smoothed response profiles with SEM indicated in shading. (**e**) Box plots showing the firing of the same Purkinje cells as in *D* during the second half of the CS-US interval before and after the injection (as a % of baseline, left-side panel; as absolute firing rate, right-side panel). Boxplots show the median, quartiles, minimum, maximum and outliers. (**f**) Individual Purkinje cell responses to the CS before and 5 min after the injection. All data plots generated with MATLAB version 9.1.0 (R2016b), Natick, Massachusetts: The MathWorks Inc.

In 16 decerebrate ferrets, we made extracellular recordings of 30 Purkinje cells from a blink-controlling area in the C3 zone of lobule HVI, identified by short-latency complex spike responses to electrical periocular stimulation^15,28^. The Purkinje cells had been trained with electrical stimulation of the ipsilateral forelimb as the CS (50 Hz, 400 or 450 ms) and direct electrical stimulation of climbing fibers (two stimulus trains of five pulses each at 500 Hz separated by 10 ms) with a delay of 150–450 ms as the US (Fig. 1a)^13^. When the Purkinje cells responded to the CS with characteristic pause CRs (Fig. 1a), usually after 2–3 h of conditioning, drugs were applied in a nanoliter range (~ 0.5–1.5 nl) injected 15 μm from the tip of the recording electrode.

First, a low concentration (5 μM) of the K_ir_3 antagonist Tertiapin_LQ_ was applied near 6 Purkinje cells that had been conditioned with a 300 ms CS-US interval (example cell in Fig. 1b). A considerably increased spontaneous firing rate or otherwise altered firing behavior could plausibly be disruptive for the expression of a Purkinje cell CR. Importantly, analyses of inter-spike intervals revealed that spontaneous firing was not affected. The average firing frequency, inter-spike interval median and maximum firing rate (a measure of spike bursting defined as the reciprocal of the shortest inter-spike interval that accounted for at least 5% of inter-spike intervals) were unchanged (Fig. 1c). Nor were there any changes in the coefficients of “global” variance (CV: 2.2 ± 0.5 vs. 2.1 ± 0.3, n.s.) or “local” variance (CV2: 0.59 ± 0.04 vs. 0.60 ± 0.04, n.s.).

We then compared the CRs over 20 trials before injection and 80 trials after injection. Purkinje cell firing in vivo is naturally variable across trials and the CR is a relative decrease in firing during the CS-US interval. Therefore, conditioned responses are typically examined as normalized to the firing rate in the 600 ms preceding the onset of the CS^13^. A larger number of trials post-injection were chosen because the time course of the effects of Tertiapin_LQ_ in this setup was unknown. This analysis revealed a distinct suppression of the pause CR (Fig. 1d). As the pause CR is a delayed-onset reduction in simple spike firing most of the response consistently occurs in the second half of the CS-US interval (black trace in Fig. 1d). The mean normalized firing in this period changed from 11 to 47% of background firing (Fig. 1e, left-side panel, Wilcoxon matched-pairs signed rank test, W = − 21, *p* = 0.03). The mean absolute firing rate in the same period changed from 5 to 21 Hz (Fig. 1e, right-side panel, W = − 21, *p* = 0.03). While the CRs were not eliminated they were clearly disrupted.

The individual cell traces in Fig. 1f show the responses to the CS before and 5 min after injection. These suggest that the drug fairly rapidly has an effect on the CR. Further, note that pre-existing excitatory components during CS presentation remain similar and that there is no increase in firing at the end of the 400 ms duration CS, beyond the 300 ms CS-US interval (no US stimulation on probe trials). This suggests that there is no drastic effect on glutamate release from the parallel fibers due to suppressed pre-synaptic K_ir_3 function.

**Figure 2:**
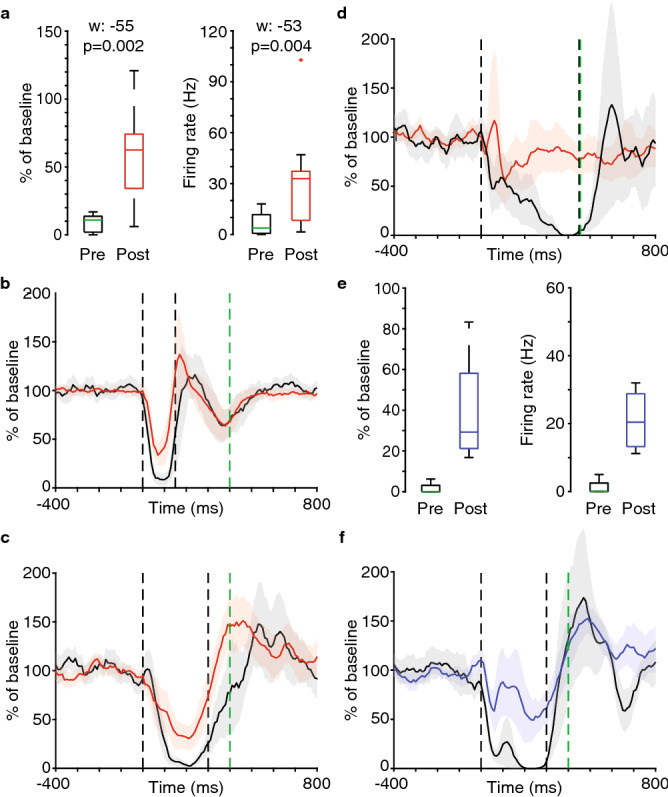
Effects of 25 μM and 200 μM doses of Tertiapin_LQ_ on conditional Purkinje cell responses. (**a**) Box plots showing the firing of Purkinje cells during the pause period of the CS-US interval before and after injection of 25 μM Tertiapin_LQ_ (*n* = 10; as a % of baseline, left-side panel; absolute firing rate, right-side panel). The pause period is the second half of the CS-US interval except for the long 450 ms interval (n = 3) where Purkinje cells only reliably paused in the last 1/3 (last 150 ms). (**b**–**d**) Average response profiles to the CS before (black) and after (red) injection of 25 μM Tertiapin_LQ_. (*B*: *n* = 2, 150 ms CS-US interval; *C*: *n* = 5, 300 ms CS-US interval; *D*: *n* = 2, 450 ms CS-US interval). (**e**) Box plots showing the firing of Purkinje cells during the second half of the CS-US interval before and after injection of 200 μM Tertiapin_LQ_ (*n* = 4; as a % of baseline, left-side panel; absolute firing rate, right-side panel). (**f**) Average response profile to the CS before (black) and after (blue) injection of 200 μM Tertiapin_LQ_ (*n* = 4, 300 ms CS-US interval). All data plots generated with MATLAB version 9.1.0 (R2016b), Natick, Massachusetts: The MathWorks Inc.

Next, we proceeded with applying mid (25 μM, *n* = 10) and high (200 μM, *n* = 4) concentrations of the drug. In the 25 μM group the mean normalized firing during the pause period changed from 9 to 58% of background (Fig. 2a, left-side panel, W = − 55, *p* = 0.002) and the absolute firing changed from 6 to 32 Hz (Fig. 2a, right-side panel, W = − 53, *p* = 0.004). As a simple control that the effect of the drug is not specific to a particular CS-US interval, this group was divided into three different CS-US intervals (150 ms, *n* = 2; 300 ms, *n* = 5; 450 ms, *n* = 3). The response profiles in Fig. 2b–d does not suggest that the effect is specific to the standard 300 ms CS-US interval. The spontaneous firing of the Purkinje cell was not affected with this higher dose (*n* = 10, firing rate: 48 vs 46 Hz, median interspike interval: 0.013 vs 0.012 s, max frequency 206 vs 215 Hz, all n.s.).

In the 200 μM group the mean normalized firing during the pause period changed from 2 to 59% of background (Fig. 2e, left-side panel) and the absolute firing changed from 1 to 21 Hz (Fig. 2e, right-side panel). The response profile before and after injection is shown in Fig. 2f. In this small group with 40 × the original dose of Tertiapin_LQ_ there were still no profound effects on spontaneous firing (firing rate: 43 vs 39 Hz, median interspike interval: 0.012 vs 0.012 s, max frequency 206 vs 252 Hz). These numbers suggest no change to 95% of spontaneous spikes (identical median and similar average) and a moderate increase in the rate when firing in bursts.

In a limited number of subjects, we also sampled Purkinje cell CRs before and after injection of antagonists of the three calcium-activated potassium channels known to be expressed by Purkinje cells (BK/K_Ca_1.1, SK/K_Ca_2.2 and IK/K_Ca_3.1 channels). This was done as a simple control for the possibility that the previously described observations with a K_ir_3.1 antagonist could be due to some general phenomenon of disrupting K^+^ functions.

**Figure 3:**
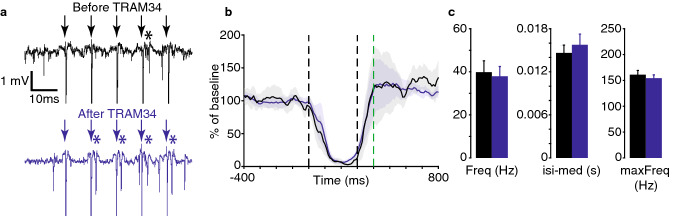
Effects of the K_ca_3.1 antagonist TRAM34 on conditional Purkinje cell responses. (**a**) An example Purkinje cell’s response to sub-threshold parallel fiber stimulation before (top) and after (bottom) injection of 1 μM TRAM34. Arrows indicate stimulation artifacts and asterisks indicate elicited simple spikes. (**b**) Population average (*n* = 4) of Purkinje cell responses to the CS (no US on test trials) before (black) and after (blue) injection of 1 μM TRAM34. (**c**) Statistics of spontaneous firing before (black) and after (blue) the injection. All data plots generated with MATLAB version 9.1.0 (R2016b), Natick, Massachusetts: The MathWorks Inc.

To establish an effective concentration of the selective K_Ca_3.1 antagonist TRAM34 we first stimulated parallel fibers directly with five pulses at 100 Hz at (5–15 μA), such that we obtained a spike response to one stimulus pulse with a probability of < 100%. Consistent with the suggested function of K_Ca_3.1 channels to suppress temporal summation of excitatory inputs^29^, TRAM34 (1 μM) increased the probability of a spike response to the second to fifth parallel fiber stimulus pulse by 156% to 202% of control (*n* = 4, example cell shown in Fig. 3a). There was no change in the Purkinje cell CR (Fig. 3b). The background firing statistics are shown in Fig. 3c.

**Figure 4:**
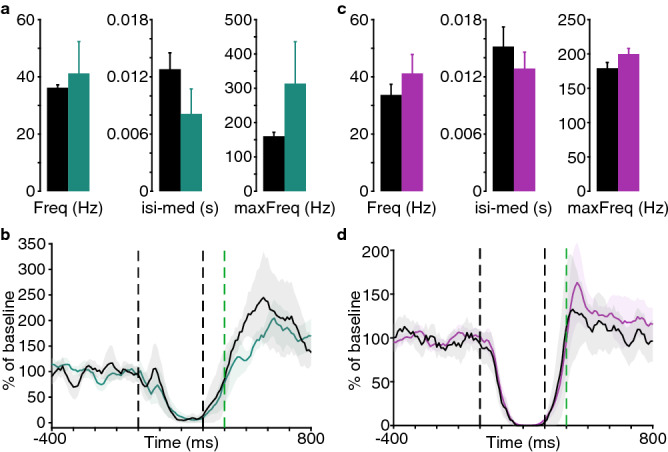
Effects of K_ca_1.1 and K_ca_2.2 antagonists on conditional Purkinje cell responses. (**a**) Statistics of spontaneous firing before (black) and after (green) injection of a K_Ca_1.1 antagonist (*n* = 3, 60 nM Penitrem A). (**b**) Average responses to the CS (no US on test trials) of the same Purkinje cells as in *A* before and after the injection. (**c**) Statistics of spontaneous firing before (black) and after (purple) injection of a K_Ca_2.2 antagonist (*n* = 4, 1 μM Apamin). (**d**) Average responses to the CS (no US on test trials) of the same Purkinje cells as in *C* before and after the injection. All data plots generated with MATLAB version 9.1.0 (R2016b), Natick, Massachusetts: The MathWorks Inc.

Consistent with previously published in vitro findings^30,31^, applying a K_Ca_1.1 antagonist (60 nM Penitrem A, *n* = 3) introduced bursting at several hundred Hz and a decrease in the median interspike interval (Fig. 4a). There was no apparent effect on the Purkinje cell CR (Fig. 4b). Upon application of a K_Ca_2.2 antagonist (1 μM Apamin, *n* = 4) we observed moderate changes in the same direction on spontaneous firing (Fig. 4c) and again no effect on the Purkinje cell CR (Fig. 4d). In summary, the observations made with K_Ca_ channel antagonists did not motivate the use of additional experimental subjects for a more systematic investigation of these three channels in the present context.

## Discussion

Our aim was to investigate whether the G-protein gated K_ir_3 channels are required for the normal expression of Purkinje cell CRs. The results suggest that they are and presents a case for G-proteins acting on potassium channels as a putative contributor to the generation of the pause in spontaneous firing during the CS-US interval.

Application of the K_ir_3 antagonist Tertiapin_LQ_ clearly weakened the normal decrease in firing in response to the CS (Fig. 1d). Any disruption of the Purkinje cell CR could in theory be a general consequence of any drug that blocks hyperpolarizing current and thus increases firing. However, such an interpretation of our findings is not justified because there were no changes in any of the measures of spontaneous firing. This suggests that were no general changes in excitability. Additionally, while our limited sampling with antagonists of calcium-activated potassium channels does not permit us to draw any major conclusions regarding their function, the collective lack of an effect on the Purkinje cell CR suggests that the observations made with the K_ir_3 antagonist is not explained by a general dysfunction of potassium channels.

As these experiments required immobilization of the subjects for tissue stability, we could not collect behavioral data. However, given the abundance of data showing that the Purkinje cell pause response drives the overt blink and precisely controls the kinematics of the blink^16,17^ we expect the effect on the overt blink to be similar.

The fact that application of the K_ir_3 antagonist did not consistently abolish all of the pause response is not surprising. This could in part be due to insufficient action of the drug on some or all of the different combinations of K_ir_3 subunits that make up the heterogenous population of tetrameric channel complexes. Perhaps more likely is that these channels are not the sole cause of the cessation in firing. Multiple intracellular signaling cascades are initiated when the CS activates G-protein receptors. It is possible that several ion channels are involved. Additionally, it is entirely possible that intracellular mechanisms are complemented by network mechanisms that in particular could contribute to setting the response amplitude, as has recently been suggessted^32,33^.

It is likely that K_ir_3 channels in parallel fiber terminals^25^ and in the axon terminals of molecular layer interneurons^34^ were also blocked in our experiments. It seems implausible that our observations are explained by the drug’s potential effect on the GABAergic interneurons. A disinhibition here would presumably lead to increased inhibitory input to the Purkinje cell, which should strengthen rather than suppress the CR. Furthermore, previous experiments have shown that ionotropic GABA receptors are not necessary for the Purkinje cell CR^14^.

We can think of two possible effects of the drug on the parallel fiber terminals. First, decreased inhibition of parallel fiber terminals and hence increased glutamate release could counteract simple spike suppression during the Purkinje cell pause response. However, in most cases the CS outlasted the ISI by 100–250 ms and if the drug caused powerful increases in glutamate release, this should be reflected in increased Purkinje cell firing after the pause duration. We observed no consistent increases in CS-elicited excitatory responses in this period (see Fig. 1d,f). Second, increased glutamate release could in theory also lead to parallel fiber synaptic fatigue such that the CS signal is not propagated to the Purkinje cell. This is unlikely because pre-existing excitatory response components, early as well as late during CS presentation, were not changed by application of the K_ir_3 antagonist (Fig. 1f).

One can also consider whether blocking K_ir_3 channels on other cell types, or compensatory mechanisms triggered by doing so, could impair the CS "representation" and therefore cause a disruption of the Purkinje cell pause response. Golgi cells constitute the most plausible potential off-target site in this scenario. While this cannot be excluded, we find it improbable for the following reasons.

First, recent in vivo data collected from the relevant region of the adult cerebellum shows that the main time constant over which Golgi cell firing has an effect on granule cells is on the order of seconds and not 10–100 ms^35^. This evident lack of fast inhibitory effects implies that the main mode of action of Golgi cells on granule cells is tonic inhibition. This would set granule cell excitability levels but would not sculpt fast temporal patterns in granule cell responses to a sensory stimulus. Consequently, we do not expect any potential effect of Tertiapin_LQ_ on the spontaneous firing of Golgi cells to impair the CS "representation" which rapidly passes through the granule and Golgi cells.

Second, once the CR has been acquired it is the initial few impulses of the CS, be it mossy fiber or forelimb stimulation, that determines the time course of the whole Purkinje cell pause^36^. Further, once conditioned, drastically varying the structure of the CS with direct parallel fiber stimulation (< 20 to 800 ms, 100–400 Hz) had no effect on the learned pause response^14^. In other words, the determining factor for triggering the CR, once it has been acquired, is essentially that the CS reaches the Purkinje cell. The records from the current experiments show that it does. While the situation could be different during the process of acquisition these tests were done on performance after acquisition. If Tertiapin_LQ_ had an unintentional off-target effect on Golgi cells which somehow altered their response to a sensory stimulus this is at the very least unlikely to have an effect on the early part of the CS.

In summary, we recognize that our experiments cannot with full certainty attribute the effects of the K_ir_3 antagonist Tertiapin_LQ_ to blockade of K_ir_3 channels exclusively on Purkinje cells. Nevertheless, to us the most parsimonious explanation of the observations is that the effect is primarily caused by blocking K_ir_3 channels on the Purkinje cells as intended.

We have previously shown that blocking mGluR7 suppresses the Purkinje cell CR^22^. While the present data show that blocking K_ir_3 also suppresses the CR we do not yet know whether a mGluR7-K_ir_3 pathway triggers the response. There is very little known about K_ir_3 activation in Purkinje cells and a GABA-B-dependent pathway is the only one thus far described^25^. Our findings raise the importance of studying possible intracellular mGluR7-K_ir_3 interactions with techniques that are not available in our setup. We also note that our experiments with mGluR7 and K_ir_3 have both concerned expression of the Purkinje cell CR once it has been acquired. Future studies that manipulate these components during the hours of the conditioning procedure would be highly valuable.

Importantly, our results do not show disrupted timing of the CRs. The reported effect is primarily a disruption of the response amplitude. We tested the idea that some non-standard type of ion channel might be involved in generating a delayed hyperpolarization and K_ir_3 was well suited in theory. While blocking these channels did not produce an effect on timing, this does not necessarily contradict a hypothesis where the timing is regulated within the Purkinje cell and, at least partially, effectuated via K_ir_3 channels. The channel antagonist used occludes the ion conduction pore from the extracellular side. It does not affect the delay introduced by the G-protein signaling cascade on the intracellular side. Therefore, an effect on the timing of the Purkinje cell response is not necessarily to be expected from application of Tertiapin_LQ_.

Selection mechanisms where the time course of the CR is regulated by selective expression or translocation of a repertoire of proteins have been proposed^20,37^. If K_ir_3 channels are confirmed to be an important part of the mechanism, changes in the channel complexes themselves or in RGS proteins are plausible candidates. The subunit composition of K_ir_3 channels determines their temporal properties^26^ and RGS proteins modulate the intrinsic GTPase activity of Gα and thus the latency to reforming of the Gαβγ complex and channel closure^24^. These components could be used to appropriately tune the pause response to the CS. Fully evaluating the hypothesis requires future experiments where these intracellular components are manipulated. Occluding the ion pore of K_ir_3 channels on the extracellular side mainly affected the amplitude with little or no effect on the timing of conditional Purkinje cell responses. Manipulating the intracellular components is expected to have a greater effect on the timing.

In conclusion, our data clearly show that application of the K_ir_3 antagonist Tertiapin_LQ_ near Purkinje cells disrupts conditional pause responses. While drug effects on other cell types cannot be excluded, our view is that the most plausible explanation of the results is that the integrity of K_ir_3 channels on the Purkinje cell is necessary for eliciting normal conditional Purkinje cell responses in classical conditioning. Importantly, this is not due to a simple increase in general excitability. The results suggest that K_ir_3 channels are legitimate candidates to explain at least part of the mechanism underlying Purkinje cell CRs. Given the prevalence of K_ir_3 channels throughout the brain, this may be a general mechanism of interest for a wide range of phenomena beyond classical conditioning.

## Methods

### Surgery

16 male one-year old ferrets (1–2 kg) were surgically prepared with electrical stimulation sites as previously described in detail^13,14^. The subjects were initially anesthetized with isoflurane, later substituted by intravenous propofol, after which they were decerebrated by sectioning the brainstem just rostral to the red nucleus. Anesthesia was then discontinued. Subjects were kept immobilized with curare and artificially ventilated. The arterial blood pressure, rectal temperature and end-expiratory CO_2_ concentration were all monitored continuously and kept within physiological limits throughout the experiment. Physiological homeostasis was maintained by intravenous infusion of 50 mg/ml glucose, isotonic acetate Ringer’s solution and Macrodex solution in proportions 1:1:1. The infusion rate was 6 ml × kg^-1^ × h^−1^. All procedures were approved by the Malmö/Lund animal research ethics committee and performed in accordance with the regulations of the Swedish Animal Welfare Act.

### Training protocol

Stimulation of cerebellar afferents during training was analogous to delay eyeblink conditioning except that the CS most often outlasted the US (see^14,18^). The CS was a 400 ms stimulus train (50 Hz, 1 ms pulse duration, 0.8–1.4 mA) applied to the ipsilateral forelimb in all but the two cells in the 450-ms CS-US interval group in Fig. 2d where the duration was 450 ms. The US consisted of two 5-pulse 500 Hz stimulus trains (0.1 ms pulse duration, 100–400 μA) separated by 10 ms, applied to ipsilateral climbing fibres in the inferior cerebellar peduncle. These parameters mimic the strongest climbing fiber response that is likely to occur under natural conditions (see methods in *13*). During paired stimulation the US was applied 150, 300 or 450 ms after CS onset. The intertrial interval was 15 ± 1 s (randomized). Acquisition sessions with paired CS-US presentations lasted 120–180 min.

### Recordings and data analysis

Extracellular recordings of Purkinje cells were performed using Carbostar-4 multibarrel electrodes (Kation Scientific, Minneapolis, U.S.A.). Purkinje cells were identified by the presence of complex spikes. Their C3 eyelid microzone identity was confirmed by short-latency climbing fiber responses to periocular stimulation^15,28^. The microelectrode signal was fed through a preamplifier and filter (Digitimer Ltd., Hertfordshire, U.K.) to a Power 1,401 data acquisition AD converter (Cambridge Electronic Design Ltd., U.K.). Off-line spike sorting was done in Spike2 (Cambridge Electronic Design Ltd., U.K.) and data analysis was done in MATLAB (MathWorks Inc., Natick, MA).

Purkinje cell CRs are constituted by decreases in firing relative to the pre-trial firing rate. Therefore, CRs are reported as a percentage of background firing. For this analysis, all spike-time data was quantified in 10-ms bins for each individual cell by averaging over 20 trials and then normalizing it relative to the 600 ms immediately preceding the CS^13^. For population plots all of the individual cell activity values where then averaged over the whole population. Raster plots show raw data and traces of cell activity in all figures are smoothed using a five point moving average. In the latter, shading indicates the standard error of the mean. Where the n is appropriate, the nonparametric Wilcoxon matched-pairs signed rank test was used for statistical analysis. The data was analyzed in the statistics toolbox G*Power (Heinrich Heine Universität Düsseldorf), which indicated an n ≥ 6 as appropriate for the test.

### Pharmacology

All ion channel antagonists were purchased from Tocris Bioscience (Bristol, UK). Stock solutions of Tertiapin_LQ_, Penitrem A and Apamin were prepared by dissolving in H_2_O and then diluted in physiological saline. TRAM34 was dissolved in DMSO and diluted in physiological saline to final DMSO concentrations of 0.1%. The drugs were kept frozen until use and injected with pressure micro-ejections through the multibarrel Carbostar electrodes. The micro-ejections were calibrated to achieve a droplet size of (0.5–1.5 nl) as described in^22^.
